# Hydrazone modification of non-food natural product sclareolide as potential agents for plant disease

**DOI:** 10.1016/j.heliyon.2022.e12391

**Published:** 2022-12-22

**Authors:** Ali Dai, Zhiguo Zheng, Yuanqin Huang, Lijiao Yu, Zhenchao Wang, Jian Wu

**Affiliations:** State Key Laboratory Breeding Base of Green Pesticide and Agricultural Bioengineering, Key Laboratory of Green Pesticide and Agricultural Bioengineering, Ministry of Education, Guizhou University, Huaxi District, Guiyang 550025, China

**Keywords:** Sesquiterpenoid derivatives, Hydrazide-hydrazone, Antiviral activity, Tobacco mosaic virus, Preliminary mechanism

## Abstract

Plant diseases and their drug resistance pose a serious threat to agricultural production. One way to solve this problem is to discover new and efficient botanical pesticides. Herein, a series of novel hydrazide-hydrazone-containing sesquiterpenoid derivatives were synthesized by simply modifying the structure of the non-food natural product sclareolide. The biological activity results illustrated that compared to ningnanmycin (39.2 μg/mL), compound **Z28** had the highest antiviral activity against tobacco mosaic virus (TMV), and the concentration for 50% of maximal effect (EC_50_) of its inactivation activity was 38.7 μg/mL, followed by compound **Z14** (40.6 μg/mL). Transmission electron microscopy (TEM) demonstrated that TMVs treated with compounds **Z14** and **Z28** were broken into rods of different lengths, and their external morphology was fragmented or even severely fragmented. Autodocking and molecular dynamics (MD) simulations indicated that compound **Z28** had a strong affinity for tobacco mosaic virus coat protein (TMV-CP), with a higher binding energy of −8.25 kcal/mol compared to ningnanmycin (−6.79 kcal/mol). The preliminary mechanism revealed that compound **Z28** can achieve an antiviral effect by targeting TMV-CP, rendering TMV unable to self-assemble and replicate, and might be a candidate for a novel plant antiviral agent. Furthermore, the curative and protective activities of compound **Z22** (EC_50_ = 16.1 μg/mL) against rice bacterial blight were 51.3% and 50.8%, respectively. Its control effect was better than that of bismerthiazol (**BT**) and thiadiazole copper (**TC**), compound **Z22** that can be optimized as an active molecule.

## 1 Introduction

**Figure 1:**
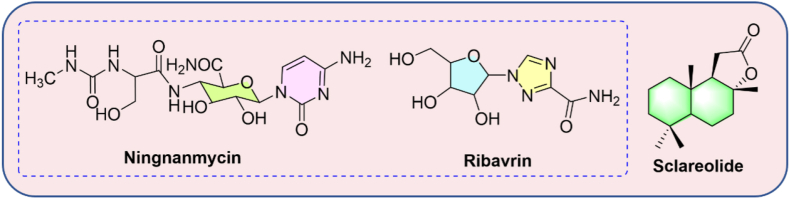
Structures of ningnanmycin, ribavirin, and sclareolide.

Plants are susceptible to infection by a variety of pathogenic microorganisms throughout their life cycle. If these microorganisms succeed in invading them and taking advantage of host-pathogen interactions, they will affect their growth and development and cause diseases [1, 2]. Plant viruses, as mandatory intracellular parasites, depend on host cells for reproduction and invasion of the host [3]. Approximately 950 plant virus diseases have been reported worldwide, causing losses of up to $30 billion every year [4]. Among them, TMV is the earliest and most well-studied virus. It has caused economic losses of up to $100 million worldwide each year due to its easy infection of tobacco, vegetables, and peppers [5, 6]. The successfully registered anti-plant virus agents, such as ningnanmycin and ribavirin (Figure 1), are widely used to prevent TMV, but their inhibition rate is low (30–60%) or the field control effect is not ideal, which results in tobacco losses [7, 8, 9]. Therefore, the research and development of novel, efficient, and low-toxic pesticides have become one of the current priorities.

As widely recognized, natural products possess novel structures, high activity, and low toxicity and have become one of the sources of new lead compounds with various biological activities [10]. Numerous compounds with good antiviral activity have been extracted from natural products, including limonoids [11], 7-Deoxy-trans-dihydronarciclasine [12], Quassinoids [13], Phenanthroindolizidines [14] *etc*. In addition, natural products or biomimetic pesticides generally have low ecotoxicological risk as well as low resistance risk [15]. Therefore, the development of new pesticides or the simple modification of their structures from natural products is an effective way to promote green pesticides and stimulate the synthesis of biomimetic pesticides.

In recent years, the use of non-food active natural products to discover potential pesticide substitutes has received extensive attention [16, 17, 18]. Sclareolide (Figure 1), a non-food bioactive drimane sesquiterpenoid, is derived from an ornamental plant – *Salvia sclarea* L., also known as clary sage, growing in temperate and subtropical climates worldwide [19]. The relevance of sesquiterpenoids for pharmaceutical and agricultural applications as well as their complex stereostructures have attracted great interest [20]. Sesquiterpenes and their derivatives possess unique biological properties for the control of insects [21], fungi/bacteria [22, 23], viruses [24, 25], inflammations [26], and sesquiterpene lactones antimalarial effect of artemisinin [27]. In particular, the sesquiterpenoids isolated [28, 29] and reported in our previous study [30] have good activity against TMV and are of great research value in commercial applications.

Meanwhile, hydrazide-hydrazone (-CO-NH-N=CH-] is an attractive multifunctional scaffold with many applications in asymmetric catalysis, coordination chemistry, pesticides, and pharmacology [31]. The structural subunits of the hydrazide-hydrazone are discovered in many compounds and exhibit a wide range of biological activities [32]. Examples include antifungal [33], antibacterial [34], antiviral [35, 36], anti-inflammatory [37], and anti-tubercular [38] activities. Moreover, the presence of nitrogen in the molecule often enhances the original activity profile of natural terpenoids [39], providing a basis for the search of new high-activity compounds.

**Figure 2:**
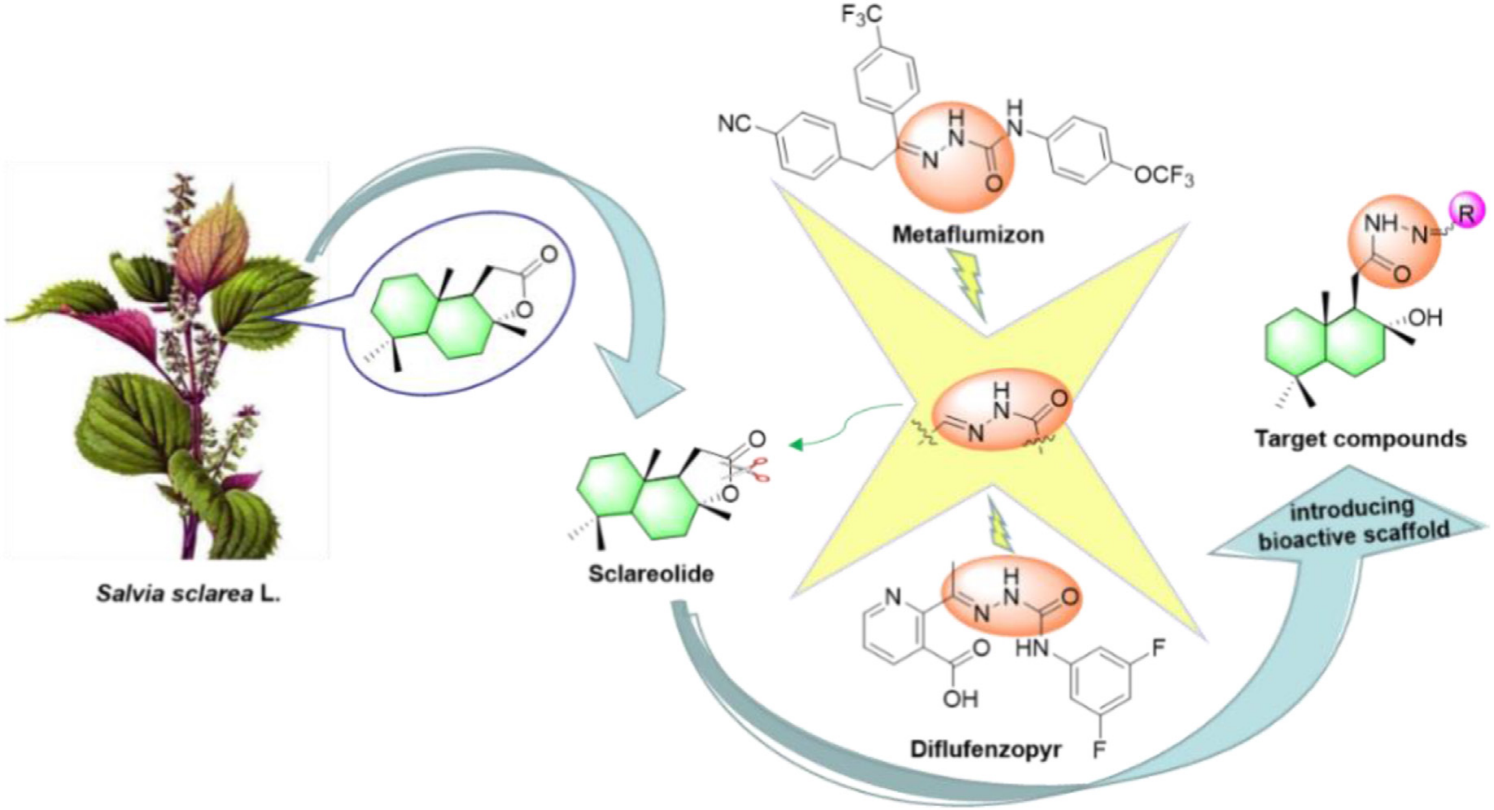
Design strategy of the target compounds.

Considering the significance of the sesquiterpene backbone and the superior properties of hydrazide-hydrazone, we continued our experiments aiming at developing new pesticides based on non-food natural products and promoting potential lead compounds as novel plant antiviral agents. Therefore, in this paper, the active fragment of hydrazide-hydrazone was introduced into sclareolide, a series of hydrazide-hydrazone-containing sesquiterpenoids were obtained by a simple synthesis in cheap and readily available absolute ethanol solution (Figure 2), and their biological activities were evaluated.

## 2 Results and discussion

### *2.1* Chemistry

**Scheme 1:**
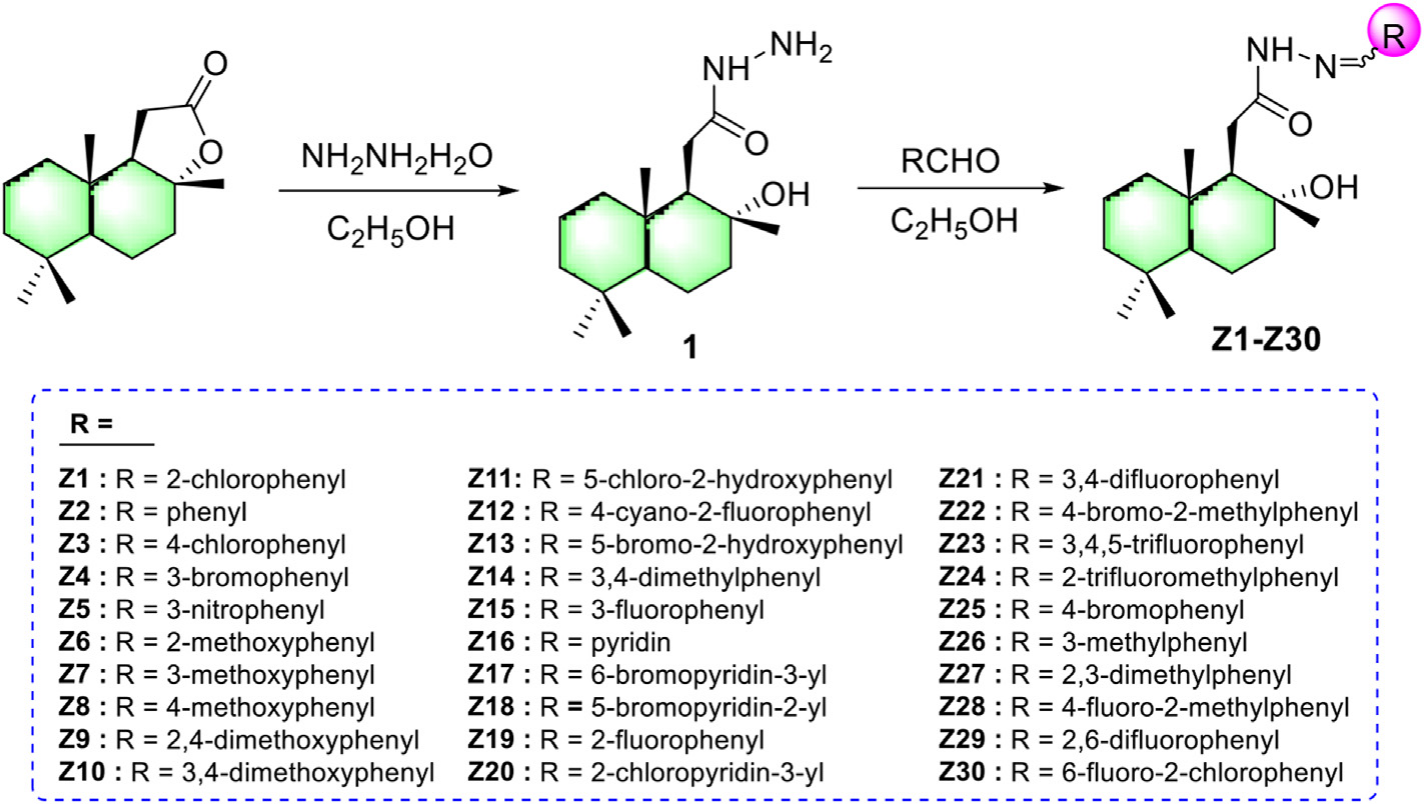
The synthetic route of the title compounds **Z1–Z30**.

The synthetic route of the title compounds **Z1–Z30** is shown in Scheme 1, in which the synthesis of the hydrazide intermediate **1** was described in our previous articles [30]. In the basic steps in the literature [40, 41], after intermediate **1** was dissolved in absolute ethanol, different aldehydes were added continuously, and the target compounds **Z1–Z30** were acquired after the reaction was completed. The structures of all compounds were identified by ^1^H NMR, ^13^C NMR, ^19^F NMR, and HRMS.

### 2.2 Biological activity

#### 2.2.1 In vitro antibacterial activity and structure-activity relationship

The preliminary antibacterial activities of the novel sesquiterpenoid derivatives **Z1–Z30** against *Xanthomonas oryzae* pv. *oryzae* (*Xoo*) and *Xanthomonas axonopodis* pv. *citri* (*Xac*) are shown in Table 1, and the EC_50_ values of some active compounds are presented in Table 2. It can be noted from Table 1 that compound **Z22** (R = 4-Br-2-CH_3_-Ph) exhibited the highest inhibition rate of 91.2% on *Xoo* at the concentration of 100 μg/mL and its EC_50_ value was 16.1 μg/mL, slightly higher than **BT** (16.2 μg/mL) and much better than **TC** (45.0 μg/mL). The activity decreased to 71.1% when R was changed to 4-Br-Ph (**Z25**) after removing –CH_3_ at position 2, but it was still higher than **TC** (65.7%). Similarly, -Br at position 4 was changed to –F (**Z28**, R = 4-F-2-CH_3_-Ph) and its activities were significantly reduced. The number and position of **F** on the benzene ring had the following order of activity towards *Xoo*: **Z15** (R = 3-F-Ph) > **Z19** (R = 2-F-Ph) > **Z21** (R = 3,4-di-F-Ph) > **Z23** (R = 3,4,5-tri-F-Ph). As shown in Table 2, the EC_50_ of compounds **Z9** (27.1 μg/mL), **Z22** (16.1 μg/mL), **Z25** (29.5 μg/mL), and **Z27** (36.2 μg/mL) were all higher than that of **TC** (45.0 μg/mL).

**Table 1:** *In vitro* antibacterial activity of the target compounds against *Xoo* and *Xac*^a^.

compd.	*Xoo*		*Xac*	
	inhibition rate (%)		inhibition rate (%)	
	100 μg/mL	50 μg/mL	100 μg/mL	50 μg/mL
**Z1**	41.6 ± 1.5	24.8 ± 1.4	68.9 ± 3.0	55.4 ± 2.5
**Z2**	55.2 ± 1.1	48.1 ± 2.1	54.5 ± 2.1	49.9 ± 1.9
**Z3**	45.3 ± 4.7	35.4 ± 0.9	36.5 ± 4.2	34.3 ± 1.3
**Z4**	54.3 ± 3.3	48.1 ± 4.1	53.0 ± 1.6	43.2 ± 2.5
**Z5**	59.9 ± 4.0	52.3 ± 2.2	64.2 ± 2.3	61.6 ± 3.8
**Z6**	17.0 ± 3.8	15.3 ± 4.9	56.6 ± 2.1	53.8 ± 2.3
**Z7**	37.1 ± 4.4	36.6 ± 4.4	81.0 ± 1.5	63.2 ± 3.8
**Z8**	46.0 ± 2.7	41.9 ± 1.3	71.0 ± 3.2	61.7 ± 0.8
**Z9**	81.5 ± 1.4	60.0 ± 4.3	70.0 ± 4.0	55.8 ± 4.2
**Z10**	53.8 ± 4.2	42.0 ± 2.0	73.4 ± 0.8	70.0 ± 3.0
**Z11**	50.8 ± 3.3	45.1 ± 0.8	47.7 ± 1.6	44.2 ± 3.2
**Z12**	16.6 ± 2.1	13.5 ± 2.9	73.4 ± 2.5	65.6 ± 1.8
**Z13**	37.7 ± 4.0	27.7 ± 2.6	37.5 ± 4.7	32.8 ± 4.2
**Z14**	34.5 ± 2.9	29.5 ± 1.7	45.5 ± 3.0	39.7 ± 1.9
**Z15**	31.3 ± 4.1	20.0 ± 1.9	62.3 ± 1.2	49.1 ± 1.5
**Z16**	19.8 ± 0.4	10.6 ± 4.2	25.3 ± 3.4	22.4 ± 2.8
**Z17**	20.7 ± 4.2	19.6 ± 1.4	27.5 ± 1.1	22.5 ± 2.5
**Z18**	35.0 ± 3.5	28.2 ± 4.7	22.7 ± 4.7	13.8 ± 4.3
**Z19**	27.1 ± 2.7	16.7 ± 4.5	40.9 ± 3.0	40.0 ± 2.7
**Z20**	38.9 ± 0.5	22.7 ± 4.2	57.4 ± 2.0	44.3 ± 3.1
**Z21**	25.9 ± 3.9	19.9 ± 2.7	68.2 ± 0.8	57.2 ± 2.1
**Z22**	91.2 ± 2.9	69.7 ± 2.3	41.1 ± 4.1	21.7 ± 1.4
**Z23**	19.9 ± 3.1	17.6 ± 3.0	45.0 ± 4.9	41.7 ± 2.1
**Z24**	54.5 ± 1.7	50.8 ± 1.8	67.8 ± 3.7	58.8 ± 1.8
**Z25**	71.1 ± 4.7	62.7 ± 0.5	57.0 ± 1.8	39.9 ± 2.2
**Z26**	35.8 ± 3.5	31.5 ± 2.9	56.3 ± 2.0	44.3 ± 3.0
**Z27**	57.8 ± 1.5	49.2 ± 3.9	39.4 ± 4.2	33.1 ± 2.5
**Z28**	25.2 ± 3.6	24.1 ± 2.8	60.2 ± 0.4	52.1 ± 4.4
**Z29**	42.6 ± 3.2	29.0 ± 2.3	49.4 ± 1.0	46.8 ± 2.1
**Z30**	44.3 ± 3.6	32.8 ± 4.8	81.3 ± 2.6	40.7 ± 3.4
**BT**^b^	88.9 ± 1.7	67.9 ± 3.0	69.6 ± 3.0	53.7 ± 4.8
**TC**^b^	65.7 ± 2.4	46.9 ± 2.7	76.8 ± 0.7	65.2 ± 2.0

a Average of three replicates.

b The commercial agricultural antibacterial agents bismerthiazol (BT) and thiadiazole copper (TC) were used as positive control.

**Table 2:** Antibacterial activities of some title compounds against *Xoo* and *Xac in Vitro*^a^.

compd.	*Xoo*			*Xac*		
	regression equation	R^2^	EC_50_ (μg/mL)	regression equation	R^2^	EC_50_ (μg/mL)
**Z1**				y = 0.98x + 3.5	0.99	31.0 ± 4.4
**Z5**	y = 0.66x + 3.8	0.99	46.4 ± 3.6			
**Z7**		0.97		y = 1.38x + 3.0	0.99	23.9 ± 0.6
**Z8**				y = 0.87x + 3.7	0.97	29.1 ± 4.3
**Z9**	y = 1.34x + 3.0	0.96	27.1 ± 1.6	y = 1.54x + 2.5	0.99	38.3 ± 3.3
**Z10**				y = 1.08x + 3.4	0.98	27.1 ± 3.0
**Z12**				y = 1.03x + 3.5	0.97	25.8 ± 4.2
**Z22**	y = 1.43x + 3.2	0.94	16.1 ± 0.7			
**Z25**	y = 1.06x + 3.4	0.96	29.5 ± 1.7			
**Z27**	y = 0.59x + 4.0	0.92	36.2 ± 4.5			
**Z30**				y = 1.21x + 3.3	0.97	22.8 ± 4.7
**BT**^b^	y = 1.62x + 3.0	0.98	16.2 ± 3.4	y = 0.83x + 3.6	0.95	46.8 ± 5.0
**TC**^b^	y = 0.93x + 3.4	0.97	45.0 ± 3.4	y = 1.04x + 3.5	0.95	23.2 ± 4.9

a Average of three replicates.

b The commercial agricultural antibacterial agents bismerthiazol (BT) and thiadiazole copper (TC) were used as positive control.

According to Tables 1 and 2, at 100 μg/mL, the antibacterial activity of compound **Z30** (R = 6-F-2-Cl-Ph) against *Xac* was 81.3%, and the EC_50_ value (22.8 μg/mL) was higher than those of **TC** (23.2 μg/mL) and **BT** (46.8 μg/mL). Compound **Z7** (R = 3-OCH_3_-Ph) possessed a good inhibitory effect on *Xac* with a slightly lower activity than the best **Z30**, and its EC_50_ (23.9 μg/mL) was close to that of **TC** and much higher than that of **BT**. When –OCH_3_ is in position 2 or 4 (e.g. **Z6**, R = 2-OCH_3_-Ph; **Z8**, R = 4-OCH_3_-Ph), its activity is reduced by 56.6% and 71.0%, respectively. When R corresponded to 3,4-di-OCH_3_-Ph (**Z10**) and 2,4-*di*-OCH_3_-Ph (**Z9**), the activities were 73.4% and 70.0%, respectively, which are better than the activity without any substituent on the benzene ring (54.5% for **Z2**). The activity of most compounds is enhanced when the H on the benzene ring is replaced by a substituent such as halogen or –OCH_3_.

#### 2.2.2 In vivo antibacterial activity

**Figure 3:**
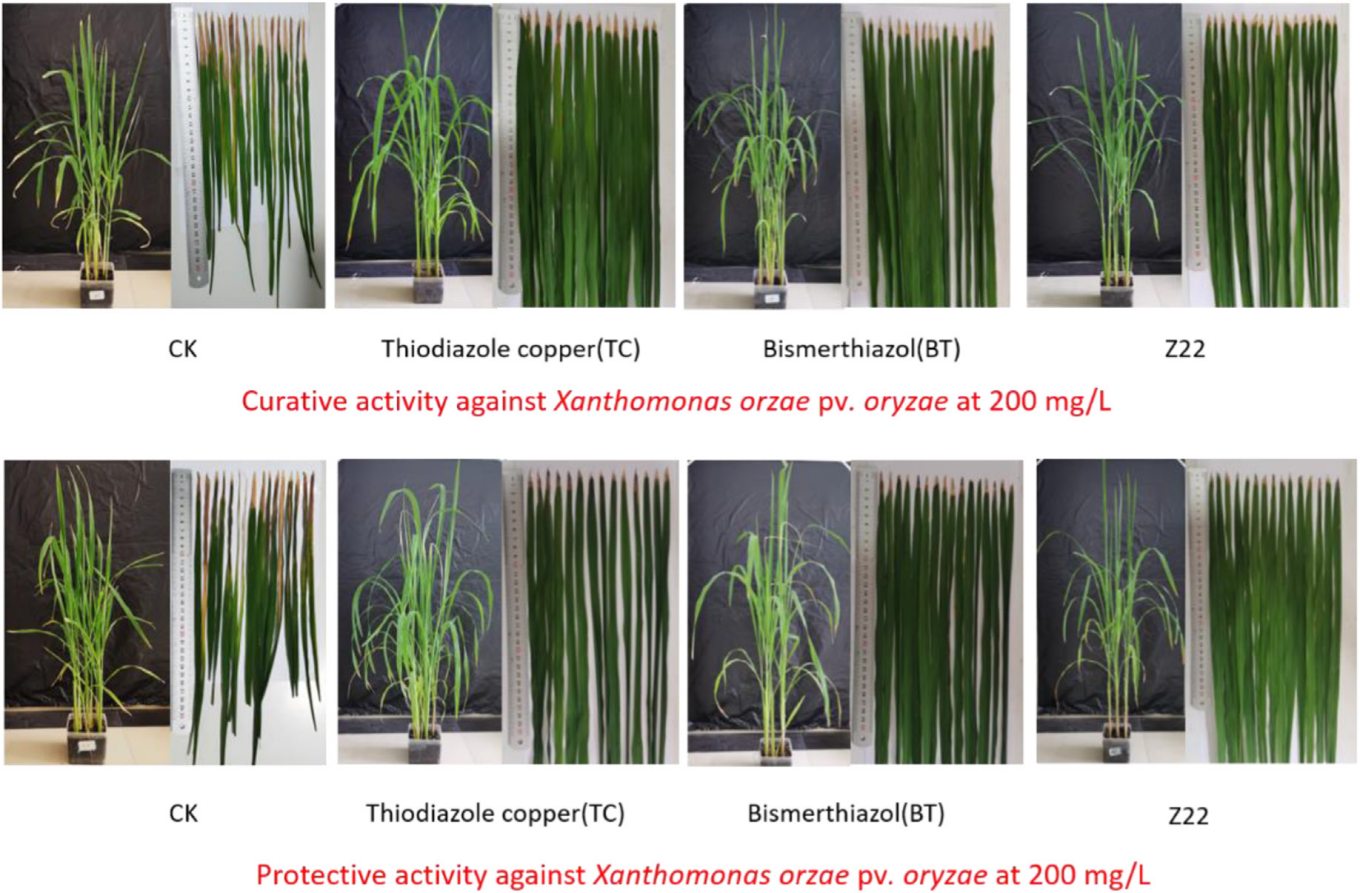
Curative and protective activities of compound **Z22** against rice bacterial leaf blight under greenhouse conditions at 200 μg/mL, with BT and TC as the positive control agents.

As can be seen above, compound **Z22** has excellent *in vitro* antibacterial activity against *Xoo*. At the concentration of 200 μg/mL, the *in vivo* antibacterial activity of compound **Z22** against rice bacterial leaf blight was determined by the leaf-cutting method. The results are shown in Tables 3 and 4, and Figure 3. The protective activity of compound **Z22** was 50.8%, which was higher than those of **BT** (45.8%) and **TC** (43.7%). Meanwhile, compound **Z22** possessed a good curative effect on rice bacterial leaf blight (51.3%), which was better than those of **BT** (47.1%) and **TC** (46.1%).

**Table 3:** The curative activity of compound Z22 against rice bacterial leaf blight.

Treatment	Curative activity (14 Days after Spraying)		
	Morbidity (%)	Disease Index (%)	Control Efficiency (%)^a^
**Z22**	100	42.2C	51.3A
**BT**^b^	100	45.8B	47.1B
**TC**^b^	100	46.6B	46.1B
**CK**^c^	100	86.7A	

a Statistical analysis was conducted by the analysis of variance method under the conditions of equal variances assumed (*P* > 0.05) and equal variances not assumed (*P* < 0.05). Different uppercase letters indicate the values of curative activity with significant difference among different treatment groups at *P* < 0.05.

b Commercial bactericides bismerthiazol (BT) and thiadiazole copper (TC) were used as positive control agents.

c Negative control.

**Table 4:** The protective activity of compound Z22 against rice bacterial leaf blight.

Treatment	Protective activity (14 Days after Spraying)		
	Morbidity (%)	Disease Index (%)	Control Efficiency (%)^a^
**Z22**	100	41.7D	50.8A
**BT**^b^	100	45.8C	45.8B
**TC**^b^	100	47.6B	43.7C
**CK**^c^	100	84.6A	

a Statistical analysis was conducted by the analysis of variance method under the conditions of equal variances assumed (*P* > 0.05) and equal variances not assumed (*P* < 0.05). Different uppercase letters indicate the values of protective activity with significant difference among different treatment groups at *P* < 0.05.

b Commercial bactericides bismerthiazol (BT) and thiadiazole copper (TC) were used as positive control agents.

c Negative control.

#### 2.2.3 In vivo anti-TMV activity and structure-activity relationship

The primary screening of target compounds **Z1–Z30** for antiviral activity against TMV and the EC_50_ of some active compounds are shown in Tables 5 and 6. Accordingly, the structure-activity relationship was preliminarily analyzed.

**Table 5:** Antiviral activities of target compounds against TMV *in vivo*^a^.

Compd.	Curative activity^b^(%)	Protective activity^b^(%)	Inactivation activity^b^(%)
**Z1**	53.3 ± 0.1	62.4 ± 4.3	54.2 ± 3.2
**Z2**	39.4 ± 0.3	55.3 ± 2.1	74.5 ± 3.9
**Z3**	48.1 ± 1.9	-	52.9 ± 0.5
**Z4**	73.0 ± 4.1	45.5 ± 3.0	53.0 ± 5.0
**Z5**	54.8 ± 4.8	-	54.3 ± 4.3
**Z6**	52.6 ± 4.0	37.8 ± 0.7	72.2 ± 2.1
**Z7**	40.0 ± 0.2	39.9 ± 0.1	78.2 ± 2.7
**Z8**	75.9 ± 1.4	27.5 ± 2.9	67.7 ± 4.6
**Z9**	37.3 ± 4.2	49.8 ± 4.0	65.7 ± 2.2
**Z10**	37.4 ± 4.2	24.8 ± 4.0	84.6 ± 1.0
**Z11**	39.2 ± 1.8	54.8 ± 3.3	53.2 ± 2.2
**Z12**	55.0 ± 1.4	-	86.9 ± 4.3
**Z13**	64.4 ± 0.9	30.1 ± 0.3	77.7 ± 5.0
**Z14**	31.1 ± 4.8	43.9 ± 2.2	92.7 ± 3.3
**Z15**	33.7 ± 2.2	44.5 ± 4.7	83.1 ± 2.3
**Z16**	31.1 ± 3.5	-	76.2 ± 2.2
**Z17**	34.2 ± 0.6	-	88.5 ± 0.6
**Z18**	33.8 ± 4.6	-	89.9 ± 1.6
**Z19**	44.2 ± 2.3	59.5 ± 4.8	86.9 ± 2.2
**Z20**	32.4 ± 2.3	58.2 ± 0.1	88.1 ± 2.2
**Z21**	46.1 ± 3.5	44.4 ± 0.2	85.8 ± 4.3
**Z22**	42.0 ± 4.5	-	79.1 ± 2.5
**Z23**	58.7 ± 2.3	33.2 ± 2.3	76.6 ± 2.0
**Z24**	35.2 ± 5.0	26.3 ± 1.6	70.0 ± 2.6
**Z25**	26.9 ± 5.0	-	82.5 ± 1.1
**Z26**	35.6 ± 3.3	-	70.9 ± 0.5
**Z27**	20.2 ± 4.3	48.6 ± 4.2	78.9 ± 2.1
**Z28**	31.3 ± 4.4	46.9 ± 2.2	93.3 ± 1.1
**Z29**	55.9 ± 2.4	-	83.8 ± 3.3
**Z30**	61.7 ± 3.8	63.6 ± 3.1	63.4 ± 1.8
**Ribavirin**^c^	44.8 ± 1.2	50.0 ± 1.8	73.5 ± 1.6
**Ningnanmycin**^c^	71.5 ± 3.5	65.3 ± 2.5	93.2 ± 0.5

a Average of three replicates.

b Concentration of compounds is 500 μg/mL.

c Commercial antiviral agent ribavirin and ningnanmycin.

**Table 6:** EC_50_ of some target compounds anti-TMV activity.

Compd.	Regression equation	R^2^	EC_50_ of Inactivation Activity^a^ (μg/mL)
**Z14**	y = 1.25x + 3.0	0.97	40.6 ± 3.4
**Z17**	y = 1.25x + 2.7	0.98	57.2 ± 3.0
**Z18**	y = 1.09x + 3.1	0.94	52.2 ± 3.5
**Z20**	y = 1.27x + 2.7	0.98	59.4 ± 3.4
**Z28**	y = 1.29x + 2.9	0.99	38.7 ± 1.4
**Ningnanmycin**^b^	y = 1.37x + 2.8	0.99	39.2 ± 3.8

a Average of three replicates.

b Ningnanmycin was used as the control.

As shown in Table 5, the hydrazide-hydrazone-containing sesquiterpenoids **Z1–Z30** exhibited good curative activity (>44.8%) against TMV compared to ribavirin at 500 μg/mL. Compound **Z8** (R = 4-OCH_3_-Ph) showed the best curative effect (75.9%), better than ningnanmycin (71.5%) and much higher than ribavirin. Its activity decreased to 52.6% and 40.0% when R was changed to 2-OCH_3_-Ph (**Z6**) and 3-OCH_3_-Ph (**Z7**), respectively. Likewise, the activity of compounds **Z9** (R = 2,4-di-OCH_3_-Ph) and **Z10** (R = 3,4-*di*-OCH_3_-Ph) was significantly reduced (<40.0%). The activity of compound **Z4** (R = 3-Br-Ph) was 73.0%, second only to that of **Z8**, and the activity of compound **Z25** (R = 4-Br-Ph) dropped sharply (26.9%) when 3-Br was changed to 4-Br. Since there is only a single electron-withdrawing on the benzene ring, the sequence of compounds' activities is as follows: **Z4** (R = 3-Br-Ph) > **Z5** (R = 3-NO_2_-Ph) > **Z1** (R = 2-Cl-Ph) > **Z3** (R = 4-Cl-Ph) > **Z19** (R = 2-F-Ph) > **Z24** (R = 2-CF_3_-Ph) > **Z15** (R = 3-F-Ph) > **Z25** (R = 4-Br-Ph).

The protective activities of title compounds **Z1–Z30** against TMV are shown in Table 5. In general, most of the compounds containing chlorine atoms had better activities, such as compounds **Z30** (R = 6-F-2-Cl-Ph) and **Z1** (R = 2-Cl-Ph); their protective activities were 63.6% and 62.4% respectively, which are close to ningnanmycin (65.3%) and higher than ribavirin (50.0%). Whereas, the activities of compounds **Z11** (R = 5-Cl-2-OH-Ph) and **Z20** (R = 2-Cl-Py-3-yl) were 54.8 and 58.2%, respectively. The effect of electron donor groups on the aromatic ring on the activity is as follows: **Z2** (R = Ph) > **Z9** (R = 2,4-di-OCH_3_-Ph) > **Z14** (R = 3,4-di-CH_3_-Ph) > **Z7** (R = 3-OCH_3_-Ph) > **Z6** (R = 2-OCH_3_-Ph) > **Z8** (R = 4-OCH_3_-Ph) > **Z10** (R = 3,4-di-OCH_3_-Ph).

**Figure 4:**
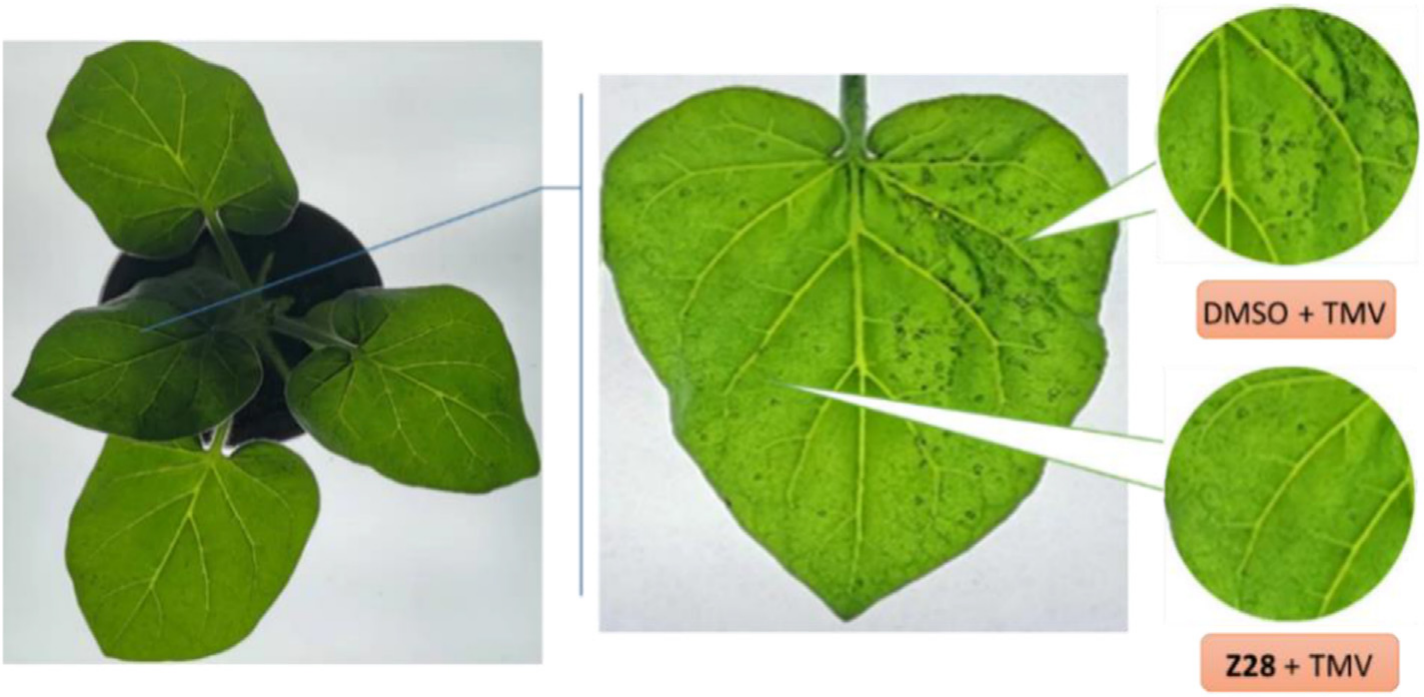
Inactivation activity *in vivo* of **Z28** against TMV.

The inactivation activities of sesquiterpenoids **Z1–Z30** on TMV are shown in Tables 5 and 6. The vast majority of the compounds had excellent inactivation activity compared to ribavirin (73.5%). Among them, the inactivation activity of compound **Z28** (R = 4-F-2-CH_3_-Ph) was 93.3% (inactivation activity *in vivo* is shown in Figure 4), and its EC_50_ was 38.7 μg/mL, which is higher than that of ningnanmycin (39.2 μg/mL). When the –F at position 4 in R was changed to -Br (**Z22**, R = 4-Br-2-CH_3_-Ph), its activity was reduced to 79.1% but still higher than that of ribavirin (73.5%). Hence, the presence of fluorine atoms on the aromatic ring increases the activity in most cases, resulting in the following order: **Z19** (R = 2-F-Ph) > **Z21** (R = 3,4-di-F-Ph) > **Z29** (R = 2,6-di-F-Ph) > **Z15** (R = 3-F-Ph) > **Z23** (R = 3,4,5-tri-F-Ph) > **Z2** (R = Ph). Furthermore, with the presence of electron-withdrawing halogen atoms on the pyridine ring, the inactivation activity was significantly improved: **Z18** (R = 5-Br-Py-2-yl) > **Z17** (R = 6-Br-Py-3-yl) > **Z20** (R = 2-Cl-Py-3-yl) > **Z16** (R = Py). The EC_50_ of compound **Z28** (38.7 μg/mL) was higher than that of ningnanmycin, while the EC_50_ of compound **Z14** (40.6 μg/mL) was close to it.

#### 2.2.4 Morphological analysis by TEM

**Figure 5:**
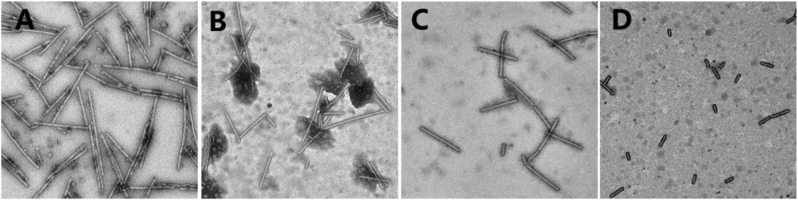
The effect on the morphology of TMV particles at 200 nm, (A) blank control, (B) ningnanmycin, (C) compound **Z14**, (D) compound **Z28**.

TEM is an indispensable means to search for possible mechanisms in the mode of action of active compounds, which can provide information such as the morphology of the TMV. Indeed, the morphology of TMV virions was observed by TEM, and the intact TMVs were found to have a rod-like structure with fewer breaks (Figure 5A). Compared to the blank control, compounds **Z28** (Figure 5D), **Z14** (Figure 5C), and ningnanmycin (Figure 5B) greatly influenced the self-assembly of TMV particles. TMVs treated with compounds **Z14**, **Z28,** and ningnanmycin were damaged, broken into rods of different lengths, and their external morphology was fragmented or even severely fragmented. The degree of fragmentation related to compound **Z14** was comparable to that of ningnanmycin, but the damage induced by compound **Z28** was the most serious. All three destroy the morphology and structure of TMV virions, and the degree of fragmentation of TMV particles is proportional to the antiviral activity. Therefore, compounds **Z14** and **Z28** may, like ningnanmycin, deprive TMV particles of their ability to infect tobacco.

#### 2.2.5 Autodocking and MD simulation

**Figure 6:**
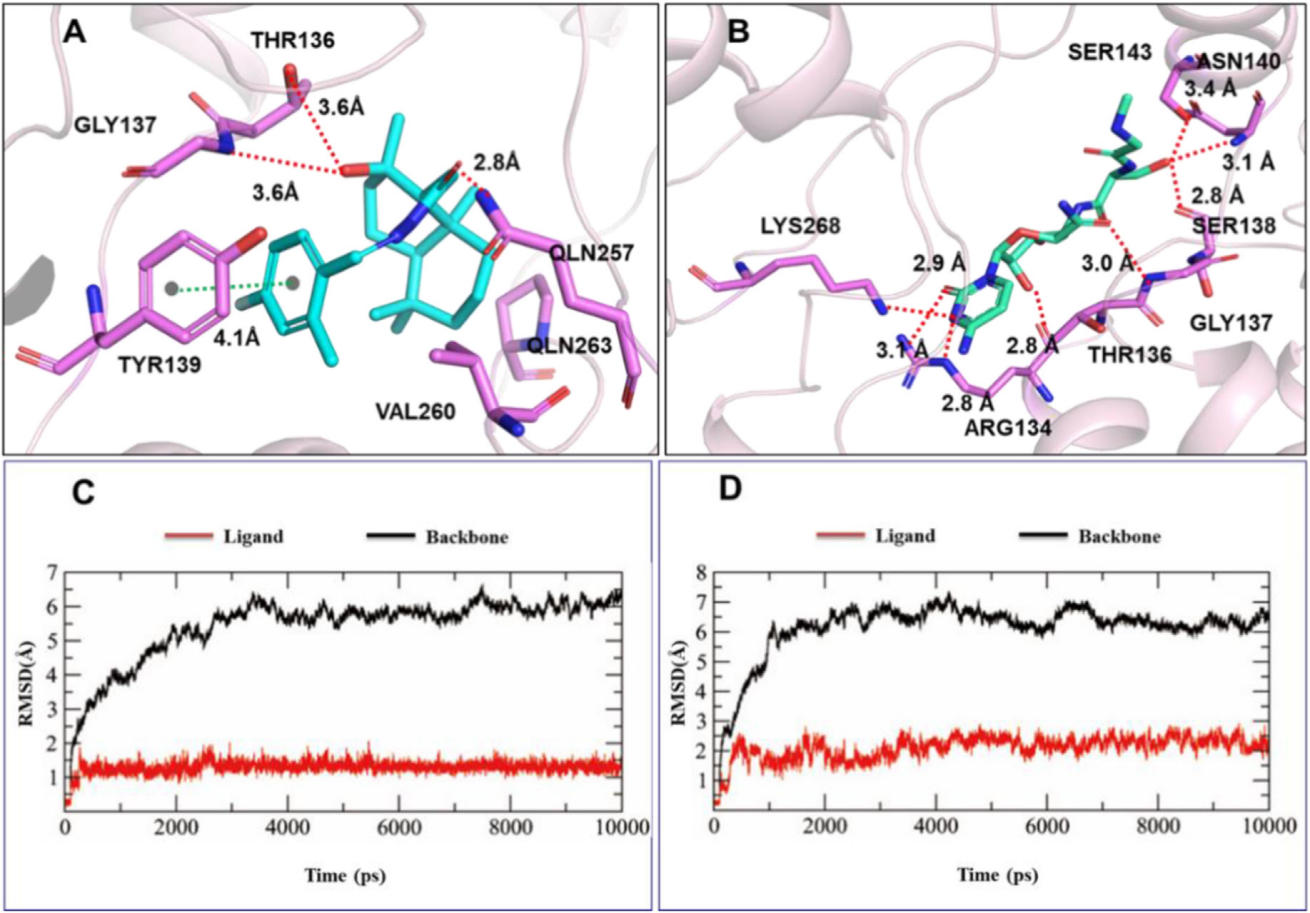
Autodocking and MD simulation studies: (A) autodocking of compound **Z28**, (B) autodocking of ningnanmycin, (C) MD simulation of compound **Z28**, (D) MD simulation of ningnanmycin.

The primary purpose of the autodocking study was to elucidate the interaction between the ligand molecules (compound **Z28** and ningnanmycin) and TMV-CP, and the results obtained are shown in Figure 6A and 6B. Compound **Z28** was inserted into the active site of TMV-CP through amino acid residues including GLY137, TYR139, THR136, QLN257, QLN263, and VAL260, which play a key role in TMV-CP self-assembly. Compound **Z28** had a strong affinity for TMV-CP with a binding energy of −8.25 kcal/mol, which is higher than −6.79 kcal/mol (ningnanmycin). In fact, there are strong hydrogen bonding interactions between the carbonyl oxygen atom, hydroxyl oxygen atom, and amino nitrogen atom of compound **Z28** and the key residues GLY137, THR136, and QLN257 with bond lengths of 3.6 Å, 3.6 Å, and 2.8 Å, respectively. The hydrocarbyl group of compound **Z28** interacts with residues TYR139, QLN263, and VAL260 via hydrophobic bonds and with TYR139 through non-covalent π-π stacking interactions. Moreover, the stability of compound **Z28** and ningnanmycin was evaluated by molecular dynamics simulation. Under simulated conditions, the root mean square deviation (RMSD) of the atoms from their initial positions was measured and recorded (Figure 6C and 6D). Due to the interaction of ligands with residues' binding sites, the energy and geometric characteristics are affected, resulting in a stable conformation and strong binding. Therefore, compound **Z28** may render TMV particles unable to self-assemble and replicate, thereby achieving an antiviral effect.

## 3 Conclusion

In summary, a series of hydrazide-hydrazone-containing sesquiterpenoid derivatives were prepared by simple modification of the non-food natural product sclareolide; the biological activity was evaluated, and the structure-activity relationship was preliminarily analyzed. The results of antiviral activity illustrated that **Z28** (EC_50_ = 38.7 μg/mL) had the best activity, which was higher than that of ningnamycin (EC_50_ = 39.2 μg/mL). Autodocking, MD simulation, and TEM studies demonstrated that the most active compound, **Z28**, can disrupt the three-dimensional structure of TMV-CP, inhibit virions' assembly, and achieve antiviral effects. Therefore, the biological activity can be influenced by optimizing the structure of the compound, and the properties of inhibiting TMV can be explored. Moreover, compound **Z22** (EC_50_ = 16.1 μg/mL) also had significant *in vivo* antibacterial activity against *Xoo*, and its control effect was better than that of bismerthiazol and thiadiazole copper, which can be optimized as a lead molecule for bactericidal activity.

## 4 Experimental sections

### 4.1 Chemistry

#### 4.1.1 General information

The synthesized hydrazide-hydrazone-containing sesquiterpenoid derivatives were characterized and their nuclear magnetic resonance (NMR, ^1^H, ^13^C, and ^19^F) spectral data were acquired on an AVANCE III HD 400 MHz (Bruker Corporation, Switzerland). Meanwhile, high-resolution mass spectrometry (HRMS) data were collected by using Thermo Scientific Q Exactive (Thermo, USA) for the analysis of the target compounds. All reactions were monitored by thin-layer chromatography and identified by UV. Melting points of all compounds were established by a specific instrument, namely the XT-4 micro melting point instrument (Beijing Tech Instrument Co., China). The FEI Talos F200C (Thermo Fisher Scientific, Waltham, MA, U.S.A.) was used for transmission electron microscopy (TEM).

All reagents and solvents were purchased from Accela ChemBio Co., Ltd (Shanghai, China) without further purification and drying. Ningnanmycin and ribavirin were sourced from Guangxi Tianyuan Biochemistry Co., Ltd. (Nanning, China), while bismerthiazol and thiadiazole copper were provided by Longwan Chemical Co., Ltd. (Zhejiang, China).

#### 4.1.2 General procedure for the synthesis of intermediate **1**

According to the general procedure in the literature [40], and the detailed synthesis procedure of intermediate **1** in our previous article [30]. A brief description of its synthesis was given, where the natural product sclareolide (500 mg, 1 mol) and hydrazine hydrate (1 mL, 11 mol) reacted by hydrazinolysis under absolute ethanol, and the precipitate collected after the treatment was intermediate **1**.

#### 4.1.3 General procedure for the synthesis of target compounds **Z1−Z30**

The title compounds were synthesized according to the method reported [41]. Intermediate **1** (300 mg, 1 mol) was dissolved in a round-bottom flask with anhydrous ethanol, and different aldehydes (150 mg, 1 mol) were added and stirred at room temperature for 6–8 h. After the reaction was completed, an appropriate amount of water was added to the system, and the precipitates were collected by filtration to obtain the target compounds **Z1**–**Z30**. The characterisation data of compounds **Z1**–**Z30** were listed as follows:

##### 4.1.3.1 N′-(2-chlorobenzylidene)-2-((1R,2R,8aS)-2-hydroxy-2,5,5,8a-tetramethyl deca hydronaphthalen-1-yl)acetohydrazide (**Z1**)

Yield 75%; White solid; m.p.83–85 °C. ^1^H NMR (400 MHz, CDCl_3_) δ 9.41 (s, 1H), 8.22 (s, 1H), 8.01–7.91 (m, 1H), 7.44–7.28 (m, 3H), 2.88 (ddd, *J* = 22.6, 16.5, 5.2 Hz, 2H), 2.10–1.92 (m, 2H), 1.87–1.73 (m, 2H), δ 1.72–1.67 (m, 1H), 1.55–1.38 (m, 2H), 1.38–1.27 (m, 2H), 1.22 (s, 3H), 1.18–1.08 (m, 1H), 1.07–0.98 (m, 2H), 0.89 (s, 3H), 0.87 (s, 3H), 0.80 (s, 3H). ^13^C NMR (100 MHz, CDCl_3_) δ 177.7, 140.3, 134.2, 131.2, 130.9, 129.9, 127.0, 126.9, 73.2, 56.9, 55.8, 44.4, 41.6, 39.3, 38.7, 33.3, 33.2, 27.6, 23.3, 21.4, 20.5, 18.4, 15.7. HRMS (ESI+) m/z Calcd for C_23_H_34_ClN_2_O_2_ [M + H]^+^ 405.23033; Found 405.23062

##### 4.1.3.2 N′-(benzylidene)-2-((1R,2R,8aS)-2-hydroxy-2,5,5,8a-tetramethyldecahydrona phthalen-1-yl)acetohydrazide (**Z2**)

Yield 93%; White solid; m.p.89–90 °C. ^1^H NMR (400 MHz, CDCl_3_) δ 9.77 (s, 1H), 7.85 (s, 1H), 7.70–7.64 (m, 2H), 7.44–7.30 (m, 3H), 2.89 (ddd, *J* = 22.4, 16.3, 5.2 Hz, 2H), 2.07–1.97 (m, 2H), 1.96–1.86 (m, 1H), 1.73–1.59 (m, 2H), 1.57–1.41 (m, 2H), 1.38–1.27 (m, 2H), 1.23 (s, 3H), 1.16–1.06 (m, 1H), 1.04–0.95 (m, 2H), 0.89 (s, 3H), 0.86 (s, 3H), 0.80 (s, 3H). ^13^C NMR (100 MHz, CDCl_3_) δ 178.1, 144.2, 133.7, 130.1, 128.7, 127.1, 73.2, 57.0, 55.8, 44.3, 41.7, 39.3, 38.8, 33.3, 33.2, 27.6, 23.5, 21.4, 20.5, 18.4, 15.6. HRMS (ESI+) m/z Calcd for C_23_H_33_N_2_O_2_ [M − H]^−^ 369.25365; Found 369.25418.

##### 4.1.3.3 N′-(4-chlorobenzylidene)-2-((1R,2R,8aS)-2-hydroxy-2,5,5,8a-tetramethyldeca hydronaphthalen-1-yl)acetohydrazide (**Z3**)

Yield 91%; White solid; m.p.94–96 °C. ^1^H NMR (400 MHz, CDCl_3_) δ 9.82 (s, 1H), 7.81 (s, 1H), 7.61–7.57 (m, 2H), 7.35 (m, 2H), 2.86 (ddd, *J* = 22.6, 16.2, 5.2 Hz, 2H), 2.02–1.83 (m, 4H), 1.74–1.64 (m, 1H), 1.60–1.44 (m, 2H), δ 1.41–1.33 (m, 2H), 1.23 (s, 3H), 1.16–1.06 (m, 1H), 1.04–0.94 (m, 2H), 0.89 (s, 3H), 0.87 (s, 3H), 0.80 (s, 3H). ^13^C NMR (100 MHz, CDCl_3_) δ 178.1, 142.8, 135.9, 132.2, 129.0, 128.3, 73.3, 57.0, 55.9, 44.4, 41.7, 39.4, 38.8, 33.3, 33.2, 27.6, 23.5, 21.4, 20.4, 18.4, 15.6. HRMS (ESI+) m/z Calcd for C_23_H_34_ClN_2_O_2_ [M + H]^+^ 405.23033; Found 405.23029.

##### 4.1.3.4 N′-(3-bromobenzylidene)-2-((1R,2R,8aS)-2-hydroxy-2,5,5,8a-tetramethyldeca hydronaphthalen-1-yl)acetohydrazide (**Z4**)

Yield 73%; White solid; m.p.83–85 °C. ^1^H NMR (400 MHz, CDCl_3_) δ 9.87 (s, 1H), 7.82 (d, *J* = 1.6 Hz, 1H), 7.79 (s, 1H), 7.58–7.27 (m, 3H), 2.86 (ddd, *J* = 22.7, 16.1, 5.3 Hz, 2H), 2.03–1.83 (m, 3H), 1.74–1.61 (m, 2H), 1.59–1.45 (m, 2H), 1.42–1.30 (m, 2H), 1.25 (s, 3H), 1.17–1.09 (m, 1H), 1.05–0.98 (m, 2H), 0.89 (s, 3H), 0.87 (s, 3H), 0.80 (s, 3H). ^13^C NMR (100 MHz, CDCl_3_) δ 178.2, 142.3, 135.8, 132.8, 130.2, 129.7, 125.9, 122.9, 73.3, 57.1, 55.9, 44.3, 41.7, 39.4, 38.8, 33.3, 33.2, 27.7, 23.6, 21.4, 20.4, 18.46, 15.6. HRMS (ESI+) m/z Calcd for C_23_H_32_BrN_2_O_2_ [M − H]^−^ 447.16417; Found 447.16599.

##### 4.1.3.5 2-((1R,2R,8aS)-2-hydroxy-2,5,5,8a-tetramethyldecahydronaphthalen-1-yl)-N′-(3-nitrobenzylidene)acetohydrazide (**Z5**)

Yield 94%; White solid; m.p.105–107 °C. ^1^H NMR (400 MHz, CDCl_3_) δ 10.11 (s, 1H), 8.49 (s, 1H), 8.25–7.95 (m, 3H), 7.62–7.47 (m, 1H), 2.89 (ddd, *J* = 22.8, 16.0, 5.4 Hz, 2H), 2.00 (m, 2H), 1.89–1.67 (m, 2H), 1.63 (m, 1H), 1.52 (m, 2H), 1.42–1.31 (m, 2H), 1.27 (s, 3H), 1.19–1.08 (m, 1H), 1.07–0.96 (m, 2H), 0.91 (s, 3H), 0.87 (s, 3H), 0.81 (s, 3H). ^13^C NMR (100 MHz, CDCl_3_) δ 178.4, 148.6, 141.2, 135.7, 132.5, 129.8, 124.3, 121.6, 73.5, 57.1, 55.9, 44.4, 41.7, 39.5, 38.8, 33.3, 33.2, 27.7, 23.6, 21.4, 20.4, 18.4, 15.6. HRMS (ESI+) m/z Calcd for C_23_H_32_N_3_O_4_ [M − H]^−^ 414.23873; Found 414.24030.

##### 4.1.3.6 2-((1R,2R,8aS)-2-hydroxy-2,5,5,8a-tetramethyldecahydronaphthalen-1-yl)-N′-(2-methoxybenzylidene)acetohydrazide (**Z6**)

Yield 98%; White solid; m.p.80–82 °C. ^1^H NMR (400 MHz, CDCl_3_) δ 9.22 (s, 1H), 8.19 (s, 1H), 7.88 (dd, *J* = 7.7, 1.6 Hz, 1H), 7.40–7.30 (m, 1H), 7.05–6.81 (m, 2H), 3.86 (s, 3H), 2.89 (ddd, *J* = 22.4, 16.4, 5.2 Hz, 2H), 2.06–1.90 (m, 4H), 1.73–1.65 (m, 1H), 1.59–1.43 (m, 2H), 1.39–1.30 (m, 2H), 1.22 (s, 3H), 1.16–1.07 (m, 1H), 1.01 (td, *J* = 12.3, 5.3 Hz, 2H), 0.89 (s, 3H), 0.86 (s, 3H), 0.80 (s, 3H). ^13^C NMR (100 MHz, CDCl_3_) δ 177.6, 158.0, 140.0, 131.4, 126.1, 122.1, 120.8, 111.0, 73.1, 56.9, 55.8, 55.5, 44.4, 41.7, 39.3, 38.8, 33.3, 33.2, 27.7, 23.4, 21.4, 20.5, 18.4, 15.6. HRMS (ESI+) m/z Calcd for C_24_H_35_N_2_O_3_ [M − H]^−^ 399.26422; Found 399.26569.

##### 4.1.3.7 2-((1R,2R,8aS)-2-hydroxy-2,5,5,8a-tetramethyldecahydronaphthalen-1-yl)-N′-(3-methoxybenzylidene)acetohydrazide (**Z7**)

Yield 95%; White solid; m.p.78–80 °C. ^1^H NMR (400 MHz, CDCl_3_) δ 9.68 (s, 1H), 7.80 (s, 1H), 7.38–7.29 (m, 1H), 7.26–7.14 (m, 2H), 6.98–6.86 (m, 1H), 3.84 (s, 3H), 2.89 (ddd, *J* = 22.4, 16.1, 5.3 Hz, 2H), 2.04–1.91 (m, 3H), 1.66 (ddd, *J* = 24.9, 17.6, 5.6 Hz, 2H), 1.58–1.42 (m, 2H), 1.35 (ddd, *J* = 22.5, 9.6, 6.6 Hz, 2H), 1.23 (s, 3H), 1.16–1.06 (m, 1H), 1.04–0.95 (m, 2H), 0.89 (s, 3H), 0.86 (s, 3H), 0.80 (s, 3H). ^13^C NMR (100 MHz, CDCl_3_) δ 178.0, 159.8, 143.9, 135.1, 129.8, 120.2, 116.1, 111.5, 73.2, 57.1, 55.8, 55.2, 44.3, 41.6, 39.3, 38.8, 33.3, 33.2, 27.7, 23.5, 21.4, 20.4, 18.4, 15.6. HRMS (ESI+) m/z Calcd for C_24_H_35_N_2_O_3_ [M − H]^−^ 399.26422; Found 399.26584.

##### 4.1.3.8 2-((1R,2R,8aS)-2-hydroxy-2,5,5,8a-tetramethyldecahydronaphthalen-1-yl)-N′-(4-methoxybenzylidene)acetohydrazide (**Z8**)

Yield 95%; White solid; m.p.91–93 °C. ^1^H NMR (400 MHz, CDCl_3_) δ 9.61 (s, 1H), 7.78 (s, 1H), 7.60 (d, *J* = 8.8 Hz, 2H), 6.92 (d, *J* = 8.8 Hz, 2H), 3.85 (s, 3H), 2.87 (ddd, *J* = 22.3, 16.2, 5.2 Hz, 2H), 2.04–1.83 (m, 3H), 1.74–1.60 (m, 2H), δ 1.58–1.43 (m, 2H)., 1.38–1.29 (m, 2H), 1.23 (s, 3H), 1.15–1.08 (m, 1H), 1.04–0.94 (m, 2H), 0.89 (s, 3H), 0.86 (s, 3H), 0.80 (s, 3H). ^13^C NMR (100 MHz, CDCl_3_) δ 177.8, 161.1, 144.0, 128.7, 126.4, 114.2, 73.1, 57.0, 55.9, 55.3, 44.3, 41.7, 39.3, 38.8, 33.3, 33.2, 27.7, 23.5, 21.4, 20.5, 18.4, 15.6. HRMS (ESI+) m/z Calcd for C_24_H_35_N_2_O_3_ [M − H]^−^ 399.26422; Found 399.26584.

##### 4.1.3.9 N′-(2,4-dimethoxybenzylidene)-2-((1R,2R,8aS)-2-hydroxy-2,5,5,8a-tetramethy ldecahydronaphthalen-1-yl)acetohydrazide (**Z9**)

Yield 95%; White solid; m.p.78–80 °C. ^1^H NMR (400 MHz, CDCl_3_) δ 9.09 (s, 1H), 8.09 (s, 1H), 7.81 (d, *J* = 8.7 Hz, 1H), 6.67–6.36 (m, 2H), 3.85 (s, 3H), 3.84 (s, 3H), 2.87 (ddd, *J* = 22.3, 16.3, 5.1 Hz, 2H), 2.07–1.90 (m, 3H), 1.71–1.61 (m, 2H), 1.59–1.43 (m, 2H), 1.39–1.29 (m, 2H), 1.21 (s, 3H), 1.12 (td, *J* = 13.1, 3.6 Hz, 1H), 1.06–0.95 (m, 2H), 0.89 (s, 3H), 0.87 (s, 3H), 0.80 (s, 3H). ^13^C NMR (100 MHz, CDCl_3_) δ 177.3, 162.6, 159.3, 140.1, 127.2, 115.1, 105.7, 98.01, 73.1, 57.0, 55.8, 55.5, 55.4, 44.4, 41.7, 39.3, 38.8, 33.3, 33.2, 27.8, 23.4, 21.4, 20.5, 18.4, 15.6. HRMS (ESI+) m/z Calcd for C_25_H_39_N_2_O_4_ [M + H]^+^ 431.29043; Found 431.28949.

##### 4.1.3.10 N′-(3,4-dimethoxybenzylidene)-2-((1R,2R,8aS)-2-hydroxy-2,5,5,8a-tetrameth yldecahydronaphthalen-1-yl)acetohydrazide (**Z10**)

Yield 93%; White solid; m.p.96–98 °C. ^1^H NMR (400 MHz, CDCl_3_) δ 9.68 (s, 1H), 7.77 (s, 1H), 7.31 (d, *J* = 1.8 Hz, 1H), 7.12 (dd, *J* = 8.3, 1.8 Hz, 1H), 6.87 (d, *J* = 8.3 Hz, 1H), 3.93 (s, 3H), 3.92 (s, 3H), 2.88 (ddd, *J* = 22.0, 15.7, 5.3 Hz, 2H), 2.06–1.91 (m, 3H), 1.68 (d, *J* = 12.7 Hz, 2H), 1.61–1.45 (m, 2H), 1.41–1.29 (m, 2H), 1.24 (s, 3H), 1.16–1.07 (m, 1H), 1.05–0.95 (m, 2H), 0.89 (s, 3H), 0.86 (s, 3H), 0.80 (s, 3H). ^13^C NMR (100 MHz, CDCl_3_) δ 177.9, 151.0, 149.2, 144.1, 126.6, 121.9, 110.7, 108.0, 73.2, 57.3, 55.9, 55.9, 55.7, 44.3, 41.6, 39.4, 38.9, 33.3, 33.2, 27.8, 23.6, 21.4, 20.4, 18.5, 15.5. HRMS (ESI+) m/z Calcd for C_25_H_37_N_2_O_4_ [M − H]^−^ 429.27478; Found 429.27628.

##### 4.1.3.11 N′-(5-chloro-2-hydroxybenzylidene)-2-((1R,2R,8aS)-2-hydroxy-2,5,5,8a-tetra methyldecahydronaphthalen-1-yl)acetohydrazide (**Z11**)

Yield 96%; White solid; m.p.138–139 °C. ^1^H NMR (400 MHz, DMSO) δ 11.59 (s, 1H), 11.30 (s, 1H), 8.29 (s, 1H), 7.60 (d, *J* = 2.7 Hz, 1H), 7.28 (dd, *J* = 8.7, 2.7 Hz, 1H), 6.93 (s, 1H), 4.14 (s, 1H), 2.51–2.50 (m, 2H), 2.18–1.86 (m, 2H), 1.77–1.70 (m, 1H), 1.60–1.38 (m, 4H), 1.37–1.27 (m, 2H), 1.14–1.08 (m, 1H), 1.00 (s, 3H), 0.93 (dd, *J* = 11.1, 5.9 Hz, 2H), 0.85 (s, 3H), 0.78 (s, 3H), 0.76 (s, 3H). ^13^C NMR (100 MHz, DMSO) δ 170.5, 156.2, 143.9, 130.8, 128.1, 123.3, 121.0, 118.5, 71.6, 56.4, 55.9, 44.0, 41.9, 38.5, 33.7, 33.3, 30.1, 24.4, 21.8, 20.5, 18.4, 15.5. HRMS (ESI+) m/z Calcd for C_23_H_33_ClN_2_O_3_Na [M + Na]^+^ 443.20719; Found 443.20544.

##### 4.1.3.12 N′-(4-cyano-2-fluorobenzylidene)-2-((1R,2R,8aS)-2-hydroxy-2,5,5,8a-tetram ethyldecahydronaphthalen-1-yl)acetohydrazide (**Z12**)

Yield 92%; White solid; m.p.98–100 °C. ^1^H NMR (400 MHz, CDCl_3_) δ 9.94 (s, 1H), 8.02 (dd, *J* = 15.9, 8.3 Hz, 2H), 7.51–7.32 (m, 2H), 2.89–2.49 (m, 2H), 2.07–1.93 (m, 3H), 1.66 (ddd, *J* = 17.0, 9.7, 2.7 Hz, 2H), 1.50–1.37 (m, 2H), 1.37–1.25 (m, 2H), 1.22 (s, 3H), 1.12 (td, *J* = 13.4, 4.0 Hz, 1H), 1.05–0.97 (m, 2H), 0.89 (s, 3H), 0.88 (s, 3H), 0.81 (s, 3H). ^13^C NMR (100 MHz, CDCl_3_) δ 178.0, 160.2 (d, *J* = 255.3 Hz), 134.6 (d, *J* = 4.3 Hz), 128.2 (d, *J* = 3.7 Hz), 127.3 (d, *J* = 3.2 Hz), 126.7 (d, *J* = 10.0 Hz), 119.8 (d, *J* = 24.5 Hz), 117.3, 114.0 (d, *J* = 9.7 Hz), 73.4, 57.0, 55.9, 44.5, 41.6, 39.4, 38.7, 33.3, 33.2, 27.5, 23.3, 21.4, 20.5, 18.4, 15.7. ^19^F NMR (376 MHz, CDCl_3_) δ −117.56. HRMS (ESI+) m/z Calcd for C_24_H_33_FN_3_O_2_K [M + K]^+^ 452.21101; Found 452.21036.

##### 4.1.3.13 N′-(5-bromo-2-hydroxybenzylidene)-2-((1R,2R,8aS)-2-hydroxy-2,5,5,8a-tetr amethyldecahydronaphthalen-1-yl)acetohydrazide (**Z13**)

Yield 96%; White solid; m.p.149–151 °C. ^1^H NMR (400 MHz, DMSO) δ 11.58 (s, 1H), 11.31 (s, 1H), 8.28 (s, 1H), 7.72 (d, *J* = 2.5 Hz, 1H), 7.39 (dd, *J* = 8.7, 2.5 Hz, 1H), 6.87 (d, *J* = 8.8 Hz, 1H), 4.13 (s, 1H), 2.54–2.44 (m, 2H), 2.17–1.87 (m, 2H), 1.76–1.70 (m, 1H), 1.59–1.38 (m, 4H), 1.36–1.29 (m, 2H), 1.14–1.07 (m, 1H), 1.00 (s, 3H), 0.93 (dd, *J* = 11.0, 5.6 Hz, 2H), 0.85 (s, 3H), 0.78 (s, 3H), 0.76 (s, 3H). ^13^C NMR (100 MHz, DMSO) δ 170.5, 156.6, 143.7, 133.6, 131.0, 121.6, 119.0, 110.7, 71.6, 56.4, 55.9, 44.0, 41.9, 38.5, 33.7, 33.3, 30.1, 24.5, 21.8, 20.5, 18.4, 15.5. HRMS (ESI+) m/z Calcd for C_23_H_32_BrN_2_O_3_ [M − H]^−^ 463.15908; Found 463.16080.

##### 4.1.3.14 N′-(3,4-dimethylbenzylidene)-2-((1R,2R,8aS)-2-hydroxy-2,5,5,8a-tetrameth yldecahydronaphthalen-1-yl)acetohydrazide (**Z14**)

Yield 95%; White solid; m.p.104–106 °C. ^1^H NMR (400 MHz, CDCl_3_) δ 9.56 (s, 1H), 7.76 (s, 1H), 7.54–7.34 (m, 2H), 7.16 (d, *J* = 7.7 Hz, 1H), 2.88 (ddd, *J* = 22.4, 16.1, 5.3 Hz, 2H), 2.29 (s, 6H), 2.03–1.92 (m, 3H), 1.72–1.62 (m, 2H), 1.59–1.43 (m, 2H), 1.40–1.29 (m, 2H), 1.24 (s, 3H), 1.11 (td, *J* = 13.1, 3.4 Hz, 1H), 1.01 (ddd, *J* = 9.9, 8.2, 6.1 Hz, 2H), 0.89 (s, 3H), 0.86 (s, 3H), 0.80 (s, 3H). ^13^C NMR (100 MHz, CDCl_3_) δ 177.9, 144.4, 139.2, 137.0, 131.3, 130.0, 128.3, 124.8, 73.2, 57.1, 55.8, 44.3, 41.7, 39.4, 38.8, 33.3, 33.2, 27.8, 23.6, 21.4, 20.4, 19.8, 19.8, 18.4, 15.6. HRMS (ESI+) m/z Calcd for C_25_H_39_N_2_O_2_ [M + H]^+^ 399.30060; Found 399.30038.

##### 4.1.3.15 N′-(3-fluorobenzylidene)-2-((1R,2R,8aS)-2-hydroxy-2,5,5,8a-tetramethyldec ahydronaphthalen-1-yl)acetohydrazide (**Z15**)

Yield 96%; White solid; m.p.86–88 °C. ^1^H NMR (400 MHz, CDCl_3_) δ 9.87 (s, 1H), 7.83 (s, 1H), 7.44–7.32 (m, 3H), 7.13–7.00 (m, 1H), δ 2.87 (ddd, *J* = 22.7, 16.3, 5.3 Hz, 2H), 2.05–1.91 (m, 3H), 1.73–1.60 (m, 2H), 1.56–1.40 (m, 2H), 1.39–1.28 (m, 2H), 1.24 (s, 3H), 1.16–1.07 (m, 1H), 1.04–0.96 (m, 2H), 0.89 (s, 3H), 0.86 (s, 3H), 0.80 (s, 3H). ^13^C NMR (100 MHz, CDCl_3_) δ 178.2, 162.9 (d, *J* = 246.4 Hz), 142.7, 136.0 (d, *J* = 7.8 Hz), 130.3 (d, *J* = 8.2 Hz), 123.4 (d, *J* = 2.6 Hz), 117.0 (d, *J* = 21.6 Hz), 113.0 (d, *J* = 22.8 Hz), 73.3, 57.0, 55.9, 44.4, 41.7, 39.4, 38.8, 33.3, 33.2, 27.6, 23.5, 21.4, 20.4, 18.4, 15.6. ^19^F NMR (376 MHz, CDCl_3_) δ −112.45. HRMS (ESI+) m/z Calcd for C_23_H_32_FN_2_O_2_ [M − H]^−^ 387.24423; Found 387.24506.

##### 4.1.3.16 2-((1R,2R,8aS)-2-hydroxy-2,5,5,8a-tetramethyldecahydronaphthalen-1-yl)-N′-(pyridin-4-ylmethylene)acetohydrazide (**Z16**)

Yield 98%; White solid; m.p.120–122 °C. ^1^H NMR (400 MHz, DMSO) δ 11.42 (s, 1H), 8.63 (s, 2H), 7.95 (s, 1H), 7.73–7.53 (m, 2H), 4.06 (s, 1H), 2.73 (ddd, *J* = 20.1, 16.5, 5.3 Hz, 1H), 2.13 (dddd, *J* = 42.4, 11.3, 10.0, 3.1 Hz, 2H), 1.77–1.43 (m, 4H), 1.38–1.22 (m, 4H), 1.17–1.08 (m, 1H), 1.06 (s, 3H), 0.97–0.91 (m, 2H), 0.85 (s, 3H), 0.83 (s, 3H), 0.77 (s, 3H). ^13^C NMR (100 MHz, DMSO) δ 176.4, 150.7, 142.9, 139.7, 121.3, 71.6, 56.4, 55.9, 44.1, 41.9, 38.5, 33.7, 33.3, 30.4, 27.0, 24.7, 21.8, 20.5, 18.4, 15.6. HRMS (ESI+) m/z Calcd for C_22_H_33_N_3_O_2_ [M + K]^+^ 412.21228; Found 412.21298.

##### 4.1.3.17 N′-(6-bromopyridin-3-yl)methylene)-2-((1R,2R,8aS)-2-hydroxy-2,5,5,8a-tetr amethyldecahydronaphthalen-1-yl)acetohydrazide (**Z17**)

Yield 96%; White solid; m.p.111–113 °C. ^1^H NMR (400 MHz, DMSO) δ 11.37 (s, 1H), 8.62 (d, *J* = 2.3 Hz, 1H), 8.07 (dd, *J* = 23.8, 22.5 Hz, 1H), 7.97 (s, 1H), 7.72 (dd, *J* = 8.3, 4.9 Hz, 1H), 4.04 (s, 1H), 2.70 (ddd, *J* = 20.1, 16.5, 5.3 Hz, 1H), 2.12 (dddd, *J* = 43.2, 11.4, 10.1, 3.1 Hz, 2H), 1.76–1.43 (m, 4H), 1.39–1.20 (m, 4H), 1.14–1.07 (m, 1H), 1.05 (s, 3H), 0.96–0.91 (m, 2H), 0.85 (s, 3H), 0.82 (s, 3H), 0.77 (s, 3H). ^13^C NMR (100 MHz, DMSO) δ 176.2, 149.6, 142.3, 141.4, 136.7, 130.7, 128.8, 71.6, 56.4, 55.7, 44.1, 41.9, 38.5, 33.7, 33.3, 30.3, 27.1, 24.7, 21.8, 20.5, 18.4, 15.6. HRMS (ESI+) m/z Calcd for C_22_H_31_BrN_3_O_2_ [M − H]^−^ 448.15942; Found 44816107.

##### 4.1.3.18 N′-(5-bromopyridin-2-yl)methylene)-2-((1R,2R,8aS)-2-hydroxy-2,5,5,8a-tetr amethyldecahydronaphthalen-1-yl)acetohydrazide (**Z18**)

Yield 95%; White solid; m.p. 104–106 °C. ^1^H NMR (400 MHz, DMSO) δ 11.40 (s, 1H), 8.72 (t, J = 2.6 Hz, 1H), 8.10 (ddd, *J* = 50.1, 24.7, 21.8 Hz, 2H), 7.85 (dd, *J* = 8.5, 6.0 Hz, 1H), 4.06 (s, 1H), 2.71 (ddd, *J* = 20.2, 16.5, 5.3 Hz, 1H), 2.13 (dddd, *J* = 40.9, 11.4, 10.0, 3.1 Hz, 2H), 1.77–1.45 (m, 4H), 1.37–1.21 (m, 4H), 1.13–1.08 (m, 1H), 1.05 (s, 3H), 0.97–0.90 (m, 2H), 0.85 (s, 3H), 0.82 (s, 3H), 0.77 (s, 3H). ^13^C NMR (100 MHz, DMSO) δ 176.2, 152.7, 150.6, 144.5, 140.0, 121.7, 120.7, 71.6, 56.5, 56.0, 44.1, 41.9, 38.5, 33.7, 33.3, 30.4, 27.0, 24.7, 21.8, 20.5, 18.4, 15.7. HRMS (ESI+) m/z Calcd for C_22_H_31_BrN_3_O_2_ [M − H]^−^ 448.15942; Found 448.16132.

##### 4.1.3.19 N′-(2-fluorobenzylidene)-2-((1R,2R,8aS)-2-hydroxy-2,5,5,8a-tetramethyldec ahydronaphthalen-1-yl)acetohydrazide (**Z19**)

Yield 94%; White solid; m.p.86–88 °C. ^1^H NMR (400 MHz, CDCl_3_) δ 9.45 (s, 1H), 8.06 (s, 1H), 7.89 (m, 1H), 7.40–7.30 (m, 1H), 7.22–6.98 (m, 2H), 2.88 (ddd, *J* = 22.5, 16.4, 5.2 Hz, 2H), 2.08–1.92 (m, 2H), 1.90–1.70 (m, 2H), 1.62–1.56 (m, 1H), 1.55–1.39 (m, 2H), 1.38–1.26 (m, 2H), 1.23 (s, 3H), 1.13 (td, *J* = 13.4, 4.0 Hz, 1H), 1.07–0.98 (m, 2H), 0.89 (s, 3H), 0.87 (s, 3H), 0.80 (s, 3H). ^13^C NMR (100 MHz, CDCl_3_) δ 177.7, 171.7, 161.3 (d, *J* = 252.3 Hz), 137.0 (d, *J* = 4.7 Hz), 131.5 (d, *J* = 8.5 Hz), 126.5 (d, *J* = 2.4 Hz), 124.4 (d, *J* = 3.5 Hz), 115.9 (d, *J* = 20.9 Hz), 73.3, 56.9, 55.8, 44.4, 41.7, 39.3, 38.7, 33.3, 33.2, 27.6, 23.3, 21.4, 20.5, 18.4, 15.6. ^19^F NMR (376 MHz, CDCl_3_) δ −120.34. HRMS (ESI+) m/z Calcd for C_23_H_32_FN_2_O_2_ [M − H]^−^ 387.24423; Found 387.24561.

##### 4.1.3.20 N′-(2-chloropyridin-3-yl)methylene)-2-((1R,2R,8aS)-2-hydroxy-2,5,5,8a-tetr amethyldecahydronaphthalen-1-yl)acetohydrazide (**Z20**)

Yield 97%; White solid; m.p.174–176 °C. ^1^H NMR (400 MHz, CDCl_3_) δ 9.90 (s, 1H), 8.45–8.16 (m, 3H), 7.32 (dd, *J* = 7.8, 4.7 Hz, 1H), 4.84 (s, 1H), 2.88–2.29 (m, 3H)., 2.00 (m, 4H), 1.75–1.49 (m, 4H), 1.23 (s, 3H), 1.16–1.09 (m, 1H), 1.06–0.98 (m, 2H), 0.88 (s, 3H), 0.88 (s, 3H), 0.80 (s, 3H). ^13^C NMR (100 MHz, CDCl_3_) δ 177.7, 150.3, 150.3, 138.6, 135.3, 128.3, 122.9, 73.4, 56.8, 55.8, 44.4, 41.6, 39.3, 38.7, 33.3, 33.2, 27.5, 23.3, 21.4, 20.5, 18.4, 15.7. HRMS (ESI+) m/z Calcd for C_22_H_31_ClN_3_O_2_ [M − H]^−^ 404.20993; Found 404.21146.

##### 4.1.3.21 N′-(3,4-difluorobenzylidene)-2-((1R,2R,8aS)-2-hydroxy-2,5,5,8a-tetramethy ldecahydronaphthalen-1-yl)acetohydrazide (**Z21**)

Yield 92%; White solid; m.p.98–100 °C. ^1^H NMR (400 MHz, CDCl_3_) δ 9.94 (s, 1H), 7.79 (s, 1H), 7.57–7.32 (m, 2H), 7.23–7.10 (m, 1H), 2.85 (ddd, *J* = 22.7, 16.3, 5.2 Hz, 2H), 2.04–1.91 (m, 3H), 1.73–1.61 (m, 2H), 1.59–1.47 (m, 2H), 1.39–1.31 (m, 2H), 1.23 (s, 3H), 1.17–1.07 (m, 1H), 1.05–0.96 (m, 2H), 0.89 (s, 3H), 0.87 (s, 3H), 0.81 (s, 3H). ^13^C NMR (100 MHz, CDCl_3_) δ 178.1, 151.5 (d, *J* = 252.8 Hz), 150.6 (d, *J* = 249.2 Hz), 141.8, 131.0 (d, *J* = 6.1 Hz), 124.0 (d, *J* = 6.5 Hz), 117.6 (d, *J* = 17.7 Hz), 115.1 (d, *J* = 18.5 Hz), 73.3, 57.0, 55.9, 44.4, 41.7, 39.4, 38.8, 33.3, 33.2, 27.6, 23.5, 21.4, 20.4, 18.4, 15.6. ^19^F NMR (376 MHz, CDCl_3_) δ −134.35, −136.51. HRMS (ESI+) m/z Calcd for C_23_H_31_F_2_N_2_O_2_ [M − H]^−^ 405.23481; Found 405.23611.

##### 4.1.3.22 N′-(4-bromo-2-methylbenzylidene)-2-((1R,2R,8aS)-2-hydroxy-2,5,5,8a-tetra methyldecahydronaphthalen-1-yl)acetohydrazide (**Z22**)

Yield 89%; White solid; m.p.88–90 °C. ^1^H NMR (400 MHz, CDCl_3_) δ 9.71 (s, 1H), 8.00 (s, 1H), 7.62 (d, *J* = 8.1 Hz, 1H), 7.36 (d, J = 8.0 Hz, 2H), 2.85 (ddd, *J* = 22.7, 16.5, 5.2 Hz, 2H), 2.44 (s, 3H), 2.03–1.84 (m, 4H), 1.72–1.66 (m, 1H), 1.61–1.50 (m, 2H), 1.38–1.30 (m, 2H), 1.21 (s, 3H), 1.14–1.05 (m, 1H), 1.02–0.96 (m, 2H), 0.87 (s, 2H), 0.87 (s, 3H), 0.80 (s, 3H). ^13^C NMR (100 MHz, CDCl_3_) δ 177.92, 141.95, 138.85, 133.81, 130.75, 129.45, 128.35, 123.92, 73.25, 56.92, 55.94, 44.46, 41.70, 39.37, 38.78, 33.35, 33.24, 27.64, 23.45, 21.43, 20.51, 19.76, 18.43, 15.68. HRMS (ESI+) m/z Calcd for C_24_H_34_BrN_2_O_2_ [M − H]^+^ 461.17982; Found 461.18115.

##### 4.1.3.23 2-((1R,2R,8aS)-2-hydroxy-2,5,5,8a-tetramethyldecahydronaphthalen-1-yl)-N′-(3,4,5-trifluorobenzylidene)acetohydrazide (**Z23**)

Yield 94%; White solid; m.p.177–179 °C. ^1^H NMR (400 MHz, CDCl_3_) δ 9.99 (s, 1H), 7.74 (s, 1H), 7.30 (dd, *J* = 10.0, 3.2 Hz, 2H), 2.84 (ddd, *J* = 22.8, 16.2, 5.3 Hz, 2H), 2.03–1.71 (m, 4H), 1.62–1.43 (m, 3H), 1.40–1.28 (m, 2H), 1.24 (s, 3H), 1.12 (td, *J* = 13.5, 4.0 Hz, 1H), 1.04–0.96 (m, 2H), 0.89 (s, 3H), 0.87 (s, 3H), 0.81 (s, 3H). ^13^C NMR (100 MHz, CDCl_3_) δ 178.2, 151.5 (d, *J* = 250.9 Hz), 151.4 (d, *J* = 251.2 Hz), 140.7 (d, *J* = 256.0 Hz), 140.6, 130.0 (d, *J* = 4.6 Hz), 111.0 (d, *J* = 6.4 Hz), 110.8 (d, *J* = 6.1 Hz).73.4, 57.0, 55.9, 44.4, 41.7, 39.5, 38.7, 33.3, 33.2, 27.6, 23.5, 21.4, 20.4, 18.4, 15.7. ^19^F NMR (376 MHz, CDCl_3_) δ −133.05, −157.04. HRMS (ESI+) m/z Calcd for C_23_H_30_F_3_N_2_O_2_ [M − H]^−^ 423.22539; Found 423.22632.

##### 4.1.3.24 2-((1R,2R,8aS)-2-hydroxy-2,5,5,8a-tetramethyldecahydronaphthalen-1-yl)-N′-(2-(trifluoromethyl)benzylidene)acetohydrazide (**Z24**)

Yield 81%; White solid; m.p.75–76 °C. ^1^H NMR (400 MHz, CDCl_3_) δ 9.85 (s, 1H), 7.91 (s, 1H), 7.89–7.81 (m, 2H), 7.66–7.40 (m, 2H), 2.88 (ddd, *J* = 22.7, 16.0, 5.4 Hz, 2H), 2.04–1.93 (m, 2H), 1.90–1.84 (m, 1H), 1.76–1.63 (m, 2H), 1.56–1.41 (m, 2H), 1.39–1.30 (m, 2H), 1.26 (s, 3H), 1.16–1.08 (m, 1H), 1.06–0.98 (m, 2H), 0.90 (s, 3H), 0.86 (s, 3H), 0.81 (s, 3H). ^13^C NMR (100 MHz, CDCl_3_) δ 178.3, 142.2, 134.6, 131.4, 130.1, 129.3, 125.1, 125.0 (q, *J* = 276.3 Hz), 73.4, 57.2, 55.9, 44.4, 41.6, 39.5, 38.8, 33.3, 33.2, 27.8, 23.5, 21.4, 20.4, 18.4, 15.6. ^19^F NMR (376 MHz, CDCl_3_) δ −62.88. HRMS (ESI+) m/z Calcd for C_24_H_32_F_3_N_2_O_2_ [M − H]^−^ 437.24104; Found 437.24207.

##### 4.1.3.25 N′-(4-bromobenzylidene)-2-((1R,2R,8aS)-2-hydroxy-2,5,5,8a-tetramethylde cahydronaphthalen-1-yl)acetohydrazide (**Z25**)

Yield 95%; White solid; m.p.122–123 °C. ^1^H NMR (400 MHz, CDCl_3_) δ 9.78 (s, 1H), 7.78 (s, 1H), 7.55–7.42 (m, 4H), 2.86 (ddd, *J* = 22.6, 16.3, 5.2 Hz, 2H), 2.02–1.90 (m, 3H), 1.73–1.47 (m, 4H), 1.42–1.32 (m, 2H), 1.23 (s, 3H), 1.16–1.06 (m, 1H), 1.04–0.94 (m, 2H), 0.89 (s, 3H), 0.87 (s, 3H), 0.80 (s, 3H). ^13^C NMR (100 MHz, CDCl_3_) δ 178.0, 142.8, 132.7, 132.0, 128.5, 124.3, 73.3, 57.0, 55.9, 44.4, 41.7, 39.4, 38.8, 33.3, 33.2, 27.6, 23.5, 21.4, 20.4, 18.4, 15.6. HRMS (ESI+) m/z Calcd for C_23_H_32_BrN_2_O_2_ [M − H]^−^ 447.16417; Found 447.16537.

##### 4.1.3.26 2-((1R,2R,8aS)-2-hydroxy-2,5,5,8a-tetramethyldecahydronaphthalen-1-yl)-N′-(3-methylbenzylidene)acetohydrazide (**Z26**)

Yield 97%; White solid; m.p.86–88 °C. ^1^H NMR (400 MHz, CDCl_3_) δ 9.70 (s, 1H), 7.81 (s, 1H), 7.47 (d, *J* = 6.4 Hz, 2H), 7.30 (d, *J* = 7.7 Hz, 1H), 7.21 (d, *J* = 7.0 Hz, 1H), 2.89 (ddd, *J* = 22.5, 16.1, 5.2 Hz, 2H), 2.38 (s, 3H), 2.03–1.92 (m, 3H), 1.66 (m, 2H), 1.60–1.45 (m, 2H), 1.40–1.31 (m, 2H), 1.24 (s, 3H), 1.15–1.08 (m, 1H), 1.05–0.98 (m, 2H), 0.89 (s, 3H), 0.86 (s, 3H), 0.80 (s, 3H). ^13^C NMR (100 MHz, CDCl_3_) δ 178.0, 144.3, 138.4, 133.6, 130.9, 128.6, 127.8, 124.4, 73.2, 57.1, 55.9, 44.3, 41.7, 39.4, 38.8, 33.3, 33.2, 27.7, 23.6, 21.4, 21.3, 20.4, 18.4, 15.6. HRMS (ESI+) m/z Calcd for C_24_H_35_N_2_O_2_ [M − H]^−^ 383.26930; found 383.27063

##### 4.1.3.27 N′-(2,3-dimethylbenzylidene)-2-((1R,2R,8aS)-2-hydroxy-2,5,5,8a-tetrameth yldecahydronaphthalen-1-yl)acetohydrazide (**Z27**)

Yield 84%; White solid; m.p.77–79 °C. ^1^H NMR (400 MHz, CDCl_3_) δ 9.65 (s, 1H), 8.16 (s, 1H), 7.65 (d, *J* = 7.1 Hz, 1H), 7.24–7.09 (m, 2H), 2.88 (ddd, *J* = 22.5, 16.4, 5.2 Hz, 2H), 2.35 (s, 3H), 2.31 (s, 3H), 2.03–1.91 (m, 3H), 1.69–1.58 (m, 2H), 1.50 (m, 2H), 1.34 (m, 2H), 1.21 (s, 3H), 1.14–1.06 (m, 1H), 1.03–0.95 (m, 2H), 0.88 (s, 3H), 0.86 (s, 3H), 0.80 (s, 3H). ^13^C NMR (100 MHz, CDCl_3_) δ 177.91, 143.55, 137.44, 135.67, 131.83, 131.51, 125.73, 124.73, 73.17, 56.98, 55.91, 44.43, 41.72, 39.34, 38.82, 33.35, 33.25, 27.73, 23.46, 21.44, 20.53, 18.44, 15.66, 15.13. HRMS (ESI+) m/z Calcd for C_25_H_37_N_2_O_2_ [M − H]^+^ 397.28495; Found 397.28598.

##### 4.1.3.28 N′-(4-fluoro-2-methylbenzylidene)-2-((1R,2R,8aS)-2-hydroxy-2,5,5,8a-tetra methyldecahydronaphthalen-1-yl)acetohydrazide (**Z28**)

Yield 98%; White solid; m.p.80–82 °C. ^1^H NMR (400 MHz, CDCl_3_) δ 9.77 (s, 1H), 8.04 (s, 1H), 7.74 (dd, *J* = 8.6, 6.0 Hz, 1H), 6.97–6.87 (m, 2H), 2.86 (ddd, *J* = 22.6, 16.5, 5.2 Hz, 2H), 2.47 (m, 3H), 2.02–1.95 (m, 3H), 1.68–1.56 (m, 2H), 1.49–1.36 (m, 2H), 1.34–1.24 (m, 2H), 1.21 (s, 3H), 1.14–1.06 (m, 1H), 1.03–0.93 (m, 2H), 0.87 (s, 3H), 0.86 (s, 3H), 0.80 (s, 3H). ^13^C NMR (100 MHz, CDCl_3_) δ 177.9, 163.3 (d, *J* = 250.3 Hz), 142.1, 139.6 (d, *J* = 8.2 Hz), 129.0 (d, J = 8.9 Hz), 128.0 (d, *J* = 3.1 Hz), 117.6 (d, *J* = 21.5 Hz), 113.4 (d, *J* = 21.7 Hz), 73.2, 56.9, 55.9, 44.4, 41.7, 39.3, 38.8, 33.3, 33.2, 27.6, 23.4, 21.4, 20.5, 20.0, 18.4, 15.6. ^19^F NMR (376 MHz, CDCl_3_) δ −110.94. HRMS (ESI+) m/z Calcd for C_24_H_34_FN_2_O_2_ [M − H]^−^ 401.25988; Found 401.26086.

##### 4.1.3.29 N′-(2,6-difluorobenzylidene)-2-((1R,2R,8aS)-2-hydroxy-2,5,5,8a-tetramethy ldecahydronaphthalen-1-yl)acetohydrazide (**Z29**)

Yield 93%; White solid; m.p.99–101 °C. ^1^H NMR (400 MHz, CDCl_3_) δ 9.66 (s, 1H), 7.97 (s, 1H), 7.34–7.28 (m, 1H), 6.97–6.88 (m, 2H), 2.88 (qd, *J* = 16.3, 5.3 Hz, 2H), 2.05–1.91 (m, 2H), 1.73–1.60 (m, 2H), 1.55–1.48 (m, 1H), 1.47–1.35 (m, 2H), 1.35–1.24 (m, 2H), 1.21 (s, 3H), 1.17–1.07 (m, 1H), 1.06–0.98 (m, 2H), 0.87 (s, 6H), 0.80 (s, 3H). ^13^C NMR (100 MHz, CDCl_3_) δ 178.2, 161.1 (d, *J* = 256.9 Hz), 161.04(d, *J* = 257.0 Hz), 133.7, 130.8 (d, *J* = 21.2 Hz), 111.9 (d, *J* = 25.1 Hz), 111.5 (d, *J* = 13.3 Hz), 73.3, 57.3, 55.8, 44.4, 41.7, 39.2, 38.7, 33.3, 33.2, 27.5, 23.1, 21.4, 20.5, 18.4, 15.5. ^19^F NMR (376 MHz, CDCl_3_) δ −134.28. HRMS (ESI+) m/z Calcd for C_23_H_33_F_2_N_2_O_4_ [M + H]^+^ 407.25046; Found 407.25162.

##### 4.1.3.30 N′-(2-chloro-6-fluorobenzylidene)-2-((1R,2R,8aS)-2-hydroxy-2,5,5,8a-tetra methyldecahydronaphthalen-1-yl)acetohydrazide (**Z30**)

Yield 94%; White solid; m.p.103–105 °C. ^1^H NMR (400 MHz, CDCl_3_) δ 9.76 (s, 1H), 8.10 (s, 1H), 7.23 (td, *J* = 7.9, 2.2 Hz, 2H), 7.08–7.02 (m, 1H), 2.88 (ddd, *J* = 22.2, 16.4, 5.1 Hz, 2H), 2.06–1.92 (m, 3H), 1.71–1.45 (m, 4H), 1.36–1.30 (m, 2H), 1.19 (s, 3H), 1.15–1.08 (m, 1H), 1.05–0.97 (m, 2H), 0.86 (s, 6H), 0.79 (s, 3H). ^13^C NMR (100 MHz, CDCl_3_) δ 178.24, 161.10 (d, *J* = 259.3 Hz), 136.65, 134.78 (d, *J* = 4.3 Hz), 130.56 (d, *J* = 9.9 Hz), 126.01 (d, *J* = 3.6 Hz), 120.39 (d, *J* = 12.5 Hz), 115.10 (d, *J* = 22.2 Hz), 73.28, 57.18, 55.87, 44.45, 41.75, 39.17, 38.79, 33.36, 33.24, 27.56, 23.22, 21.43, 20.55, 18.43, 15.55. ^19^F NMR (376 MHz, CDCl_3_) δ −109.69. HRMS (ESI+) m/z Calcd for C_23_H_31_FClN_2_O_2_ [M − H]^+^ 421.20526; Found 421.20673.

### 4.2 Biological method

#### 4.2.1 Anti-TMV activity in vivo bioassay

TMV was isolated and purified from TMV-infected tobacco in accordance with literature methods [42, 43]. The curative, protective, and inactivation activities of the title compounds were evaluated against TMV at 500 μg/mL by the half-leaf method [44]. Similarly, the half-maximal effective concentrations (EC_50_) of some compounds were calculated at 500, 250, 125, 62.5, and 31.25 *μ*g/mL, respectively. Specific details of the TMV purification and testing methods are described in the Supporting Information. The commercial antivirals ningnanmycin and ribavirin were used as positive controls and dimethyl sulfoxide (DMSO) as a negative control.

#### 4.2.2 In vitro bioassay of antibacterial activity

The *in vitro* antibacterial activities of the target compounds against two plant pathogenic bacteria *Xanthomonas oryzae* pv. *oryzae (Xoo)* and *Xanthomonas axonopodis* pv*. citri (Xac)* were tested by turbidimetric assays [45, 46] at concentrations of 100 and 50 mg/L, respectively. The commercial bactericides thiadiazole copper (**TC**) and bismerthiazol (**BT**) were used as positive controls, and an aqueous DMSO solution was used as blank control. A 1 mL aliquot of the prepared compound solution was pipetted into a test tube containing 4 mL of the medium, and then *Xoo* and *Xac* were added. Subsequently, the test tube was stirred in a shaker at a constant temperature (180 rpm, 28 ± 1 °C) until the OD value of the blank control reached 0.6–0.8, then the OD value of the compound at 595 nm was tested. The formula to calculate the inhibition rate is *I* (%): *I* = (*C* − *T*)/*C* × 100%, where *C* is the absorbance value of the blank control and *T* is the treated absorbance value.

#### 4.2.3 In vivo bioassay against rice bacterial leaf blight

On the basis of literature reports [47], the potted control effect of compound **Z22** on rice bacterial leaf blight was determined. Aqueous DMSO solution was used as blank control, while commercial bactericides thiadiazole copper (**TC**) and bismerthiazol (**BT**) were used as positive control agents. In the curative activity assay, inoculation of *Xoo* on rice leaves one day after, compound **Z22** (200 μg/mL) or aqueous DMSO solution was sprayed and the disease index of inoculated rice was determined after 14 days. To evaluate the protective effect, 200 μg/mL of compound **Z22** or aqueous DMSO solution was sprayed on rice, *Xoo* was inoculated 24 h later, and the disease index of the inoculated rice was measured after 14 days. The control efficiency *I* (%) of curative and protective activities was calculated as *I* (%) = (*C* − *T*)/*C* × 100%, where *C* is the disease index of the negative control group, and *T* is the disease index of the treatment group.

#### 4.2.4 Morphological observation by TEM

Following the general procedure in the literature [48], equal volumes of DMSO solution (blank control) and compounds solution (1000 μg/mL of ningnanmycin and active compounds) were mixed with TMV for 30 min. The mixture was adsorbed onto 200-mesh carbon-coated copper grids treated and counterstained with 1% phosphotungstic acid. After drying, the morphology of TMV particles was observed under 200 kv TEM with FEI Talos F200C.

#### 4.2.5 Autodocking and MD simulation

The crystal structure of TMV-CP (PDB code 1EI7) was downloaded from the RCSB protein database (PDB, http://www.rcsb.org) [49, 50]. Ligand molecules (ningnanmycin and active compounds) and receptors were processed, and targets were identified. Next, appropriate parameters were selected for different systems. After determining the parameters with ningnanmycin as standard, the active compound was docked on AutoDOCK-4.6 to find the optimal molecular pocket. The MD simulation was performed on Amber.

## Declarations

### Author contribution statement

Ali Dai: Performed the experiments; Analyzed and interpreted the data; Wrote the paper.

Zhiguo Zheng: Analyzed and interpreted the data; Wrote the paper.

Yuanqin Huang: Analyzed and interpreted the data.

Lijiao Yu: Performed the experiments.

Zhenchao Wang and Jian Wu: Conceived and designed the experiments; Contributed reagents, materials, analysis tools or data.

### Funding statement

Dr./Prof. Jian Wu was supported by 10.13039/501100001809National Natural Science Foundation of China [32072445 and 21762012], Program of Introducing Talents to Chinese Universities [D20023].

### Data availability statement

Data included in article/supp. material/referenced in article.

### Declaration of interest's statement

The authors declare no conflict of interest.

### Additional information

Physical characteristics, original spectral files, HRMS spectra, and ^1^H, ^13^C, ^19^F NMR spectra of all target compounds (**Z1**–**Z30)** were listed in Supporting information (SI).

Supplementary content related to this article has been published online at https://doi.org/10.1016/j.heliyon.2022.e12391.

## Appendix B Supplementary data

SI for HLY e12391_V2 SI for HLY e12391_V2
